# Human herpes virus-6 seroprevalence and leukaemias: a case-control study

**DOI:** 10.1038/sj.bjc.6690471

**Published:** 1999-06

**Authors:** G Gentile, A Mele, G Ragona, A Faggioni, C Zompetta, M E Tosti, G Visani, G Castelli, A Pulsoni, B Monarca, P Martino, F Mandelli, G I M E M A (Gruppo Italiano Malattie Ematologiiche dell' Adulto)

**Affiliations:** Dipartimento di Biotecnologie Cellulari e Ematologia, Università ‘La Sapienza’, via Benevento, 6-00161 Roma, Italia; Reparto di Epidemiologia Clinica, Istituto Superiore di Sanità, Roma, Italia; Dipartimento di Medicina Sperimentale e Patologia, Università ‘La Sapienza’, Roma, Italia; IRCCS ‘Neuromed’, Pozzilli, Italia; Istituto di Ematologia Seragnoli, Bologna, Italia; Istituto di Ematologia, Policlinico S Matteo, Pavia, Italia

**Keywords:** HHV-6, leukaemia, viral aetiology

## Abstract

The relationships between acute myeloid leukaemia (AML), acute lymphocytic leukaemia (ALL), chronic myeloid leukaemia (CML) and refractory anaemia with excess of blasts (RAEB) and human herpes virus (HHV)-6 antibody level were investigated in a multicentre case-control study. An association between increased HHV-6 seropositivity and geometric mean titre ratio with AML was shown: *P* for trend = 0.022, adjusted odds ratio 1.20, 95% confidence interval 1.07–1.33 respectively. No association was found between HHV-6 and ALL, CML or RAEB. © 1999 Cancer Research Campaign


*British Journal of Cancer* (1999) **80**(7), 1103–1106
© 1999 Cancer Research Campaign
Article no. bjoc.1999.0471

Short communication

### Human herpes virus-6 seroprevalence and leukaemias: a case-control study

**G Gentile[1], A Mele[2], G Ragona[3,4], A Faggioni[3], C Zompetta[3], ME Tosti[2], G Visani[5], G Castelli[6], A Pulsoni[1], B Monarca[1], P Martino[1], F Mandelli[1] and GIMEMA (Gruppo Italiano Malattie Ematologiche dell' Adulto)**

[1]Dipartimento di Biotecnologie Cellulari e Ematologia, Università 'La Sapienza', via Benevento, 6-00161 Roma, Italia; [2]Reparto di Epidemiologia Clinica, Istituto Superiore di Sanità, Roma, Italia; [3]Dipartimento di Medicina Sperimentale e Patologia, Università 'La Sapienza', Roma, Italia; [4]IRCCS 'Neuromed', Pozzilli, Italia; [5]Istituto di Ematologia Seragnoli, Bologna, Italia; [6]Istituto di Ematologia, Policlinico S Matteo, Pavia, Italia

**Summary** The relationships between acute myeloid leukaemia (AML), acute lymphocytic leukaemia (ALL), chronic myeloid leukaemia (CML) and refractory anaemia with excess of blasts (RAEB) and human herpes virus (HHV)-6 antibody level were investigated in a multicentre case-control study. An association between increased HHV-6 seropositivity and geometric mean titre ratio with AML was shown: *P* for trend = 0.022, adjusted odds ratio 1.20, 95% confidence interval 1.07–1.33 respectively. No association was found between HHV-6 and ALL, CML or RAEB.

**Keywords:** HHV-6; leukaemia; viral aetiology

Human herpes virus-6 (HHV-6) was first isolated from peripheral blood leucocytes of patients with lymphoproliferative disorders, including lymphoma and leukaemia (Salahuddin et al, 1986). HHV-6 is predominantly a CD4+ T-lymphotropic virus, although other cell types such as B and CD8+ T-lymphocytes, monocytes/macrophages, megakaryocytes and natural killer (NK) cells can be infected in vitro (Ablashi et al, 1988*a*; Braun et al, 1997). As for other herpesviruses, primary infection occurs mainly in childhood, being the causative agent of exanthem subitum (Braun et al, 1997). HHV-6 probably remains latent in the host after primary infection since it can be reactivated in immunocompromised patients such as those receiving a bone marrow transplant or those infected with HIV; such patients may present a broad spectrum of clinical disorders in response to infection ranging from asymptomatic infection to illnesses such as interstitial pneumonia or encephalitis (Ragona et al, 1995; Braun et al, 1997).

A role for HHV-6 in haematological malignancies, such as lymphomas has been suggested, though it is primarily a lytic virus; the transforming potential of its DNA has been demonstrated in vitro (Razzaque, 1990). Genomic DNA and several subgenomic clones from HHV-6 variant A can produce malignant transformation of NIH 3T3 cells and human keratinocytes (Razzaque, 1990; Razzaque et al, 1993; Thompson et al, 1994). HHV-6 genome integration has been found in peripheral blood mononuclear cell DNA (Luppi et al, 1993) and HHV-6 DNA sequences have been identified in the pathologic tissue of Hodgkin's disease (Di Luca et al, 1994), non-Hodgkin's lymphoma (Di Luca et al, 1994) and in peripheral blood mononuclear cells from patients with acute lymphoblastic leukaemia (ALL) (Luka et al, 1991) and with T-cell chronic lymphoproliferative disease (Braun et al, 1995).

In addition, HHV-6 seroprevalence was shown to be higher in patients with Hodgkin's disease or acute myeloid leukaemia (AML) as compared with controls (Clark et al, 1990).

*Received 26 August 1998*
*Revised 16 December 1998*
*Accepted 5 January 1999*

*Correspondence to:* G Gentile

In the present study we investigated the relationship between AML, ALL, chronic myeloid leukaemia (CML), refractory anaemia with excess of blasts (RAEB) and HHV-6 antibody level in a multicentre case-control study on risk factors for leukaemias (Mele et al, 1994).

### PATIENTS AND METHODS

This study was conducted in three Italian cities, Roma (central Italy), Bologna and Pavia (northern Italy) between 1 November 1986 and 31 March 1990.

### Recruitment of study subjects

The method of study subject recruitment and data collection is described in detail elsewhere (Mele et al, 1994). Cases were 15 years of age or older with newly diagnosed AML, ALL, CML, or RAEB. Diagnostic criteria were based on the revised French–American–British classification of bone marrow aspirates for acute leukaemias and RAEB; diagnosis for CML was based on typical and cytogenetic laboratory features. Controls were recruited during the study period among outpatients without haematological malignancies and were seen in the same hospital where cases were identified.

The control group was selected by taking the first five outpatients in Rome and the first three in Bologna and Pavia seen on a random day each week. Subjects who had received blood product transfusions were excluded from the study.

A standard precoded questionnaire on medical history, behavioural habits and environmental exposure was administered to both cases and controls. Informed consent was obtained from all study participants.

The members of GIMEMA are as follows: R Colombini (Istituto di Ematologia Seragnoli, Bologna); S Merante (Divisione di Ematologia, Pavia); A Capobianchi, S Capucci, A Vitale (Dipartimento di Biotecnologie Cellulari e Ematologia, Roma)

**Table 1** Distribution of cases and controls by HHV-6 titre

| Titres | Negative at 1:20 *n* (%) | 1:20 *n* (%) | 1:40 *n* (%) | 1:80 *n* (%) | 1:160 *n* (%) | 1:320 *n* (%) | Total *n* | Geometric means titres[a] |
|---|---|---|---|---|---|---|---|---|
| Control | 250 (29.3) | 88 (10.3) | 360 (42.2) | 115 (13.5) | 35 (4.1) | 5 (0.6) | 853 | 29.2 |
| AML | 39 (24.5) | 15 (9.4) | 65 (40.9) | 23 (14.5) | 12 (7.5) | 5 (3.1) | 159 | 35.0 |
| ALL | 16 (29.1) | 3 (5.5) | 25 (45.5) | 5 (9.1) | 5 (9.1) | 1 (1.8) | 55 | 32.3 |
| RAEB | 20 (34.5) | 1 (1.7) | 22 (37.9) | 11 (19.0) | 4 (6.9) | 0 (0.0) | 55 | 30.8 |
| CML | 31 (27.2) | 18 (15.8) | 45 (39.5) | 10 (8.8) | 8 (7.0) | 2 (1.8) | 114 | 29.9 |

[a]Sera negative at the lowest dilution have been allocated a titre of 1:10. AML, acute myeloid leukaemia; ALL, acute lymphoid leukaemia; RAEB, refractory anaemia with excess of blasts; CML, chronic myeloid leukaemia.

**Table 2** Distribution of cases and controls and geometric mean titres (GMT) by demographic characteristics

| | Control | | | AML | | | ALL | | | RAEB | | | CML | | |
|---|---|---|---|---|---|---|---|---|---|---|---|---|---|---|---|
| | *n* | GMT | *P*[a] | *n* | GMT | *P*[a] | *n* | GMT | *P*[a] | *n* | GMT | *P*[a] | *n* | GMT | *P*[a] |
| Age | | | | | | | | | | | | | | | |
| 15–19 | 41 | 30.0 | | 8 | 40.0 | | 12 | 31.8 | | 0 | – | | 1 | 10.0 | |
| 20–29 | 194 | 30.4 | | 19 | 33.3 | | 14 | 40.0 | | 1 | 10.0 | | 7 | 44.2 | |
| 30–39 | 159 | 30.8 | 0.580 | 20 | 33.6 | 0.693 | 7 | 32.8 | 0.876 | 2 | 20.0 | 0.064 | 20 | 28.3 | 0.558 |
| 40–49 | 131 | 29.6 | | 25 | 27.9 | | 3 | 31.8 | | 3 | 10.0 | | 18 | 30.5 | |
| ≥ 50 | 328 | 27.5 | | 87 | 37.5 | | 19 | 27.8 | | 52 | 34.1 | | 68 | 29.5 | |
| Sex | | | | | | | | | | | | | | | |
| Male | 313 | 29.5 | 0.749 | 75 | 31.2 | 0.212 | 22 | 29.2 | 0.513 | 36 | 31.2 | 0.895 | 71 | 28.1 | 0.355 |
| Female | 540 | 29.0 | | 82 | 37.4 | | 33 | 34.5 | | 22 | 30.1 | | 43 | 33.0 | |
| Study centre | | | | | | | | | | | | | | | |
| North | 353 | 27.1 | 0.001 | 71 | 35.6 | 0.797 | 31 | 26.7 | 0.181 | 25 | 25.2 | 0.057 | 37 | 26.4 | 0.032 |
| Centre | 500 | 32.5 | | 88 | 34.2 | | 24 | 37.4 | | 33 | 40.0 | | 77 | 38.5 | |
| Education level | | | | | | | | | | | | | | | |
| High[b] | 311 | 31.6 | 0.045 | 44 | 33.6 | 0.937 | 17 | 47.1 | 0.040 | 9 | 31.8 | 0.988 | 27 | 21.1 | 0.020 |
| Low[c] | 534 | 28.1 | | 108 | 33.2 | | 37 | 27.0 | | 46 | 31.9 | | 82 | 33.2 | |
| Total | 853 | 29.2 | | 159 | 35.0 | | 55 | 32.3 | | 58 | 30.8 | | 114 | 29.9 | |

[a]*P* for trend. [b]≥ 9 years of schooling; [c]≤ 8 years of schooling. Abbreviations as in Table 1.

### Laboratory methods

All sera were stored at – 20°C and subsequently tested. Sera were coded prior to laboratory analysis to ensure that investigators were blind to the status of each sample. Coded sera were serially diluted and tested for the presence of antibodies against HHV-6 by indirect immunofluorescence assay (IFA) on HSB-2 cells infected with HHV-6 (GS strain) as viral capsid antigen source (Ragona et al, 1994). Mock infected cells were used to evaluate non-specific background fluorescence. Antibody titres were determined by two independent investigators and expressed as the highest serum dilution yielding detectable immunofluorescence on an appreciable fraction of the antigen-positive cells.

### Statistical analysis

Geometric mean titres (GMT) were computed for each group of cases and compared with those of controls by the ratio and its 95% confidence interval (CI). In calculating GMT, sera were considered negative at the lowest dilution as 1:10. Results were similar when omitting negative sera from analysis. Multiple logistic regression was used to estimate adjusted odds ratios (OR) and 95% CI for each case group as compared with controls. BMDP software was used for all statistical analyses.

### RESULTS

Serum samples from 450 out of 636 recruited cases and from 948 out of 1193 recruited controls were tested. No differences regarding age, sex, education and leukaemia type were observed between tested and untested cases. Eighty-five controls and 74 cases who previously received blood product transfusions (e.g. blood and pooled plasma products) were excluded from the study. Thus, 386 cases enrolled at the time of leukaemia diagnosis, and 853 controls aged 15 years or older were included in this study. Iron deficiency and thalassaemia minor accounted for 43% of control diagnoses; 36% of the control group had no underlying haematological disorder.

In the control group, seropositivity at 1:80 serum dilution was placed at 82 percentile (Table 1); therefore, sera with titres at ≥ 1:80 were considered positive. The seroprevalence population was 18.2%; seroprevalence of the cases were 15.1% (AML), 20.0% (ALL), 25.5% (RAEB) and 17.6% (CML).

Cases and controls from central Italy (Roma) displayed higher seropositivity than cases and controls from northern Italy (Bologna and Pavia). Differences in GMT were found according to education level among controls, ALL and CML cases, whereas no relation with age and sex was found (Table 2).

Multiple logistic regression analysis showed that only AML presented significantly increased HHV-6 seropositivity as

 © 1999 Cancer Research Campaign

**Table 3** Odds ratios[a] by HHV-6 titre[b], *P* for trend and GMT ratios in cases and controls

| **Titres** | **1:80** | **1:160** | **1:320** | ***P* for trend** | **GMT ratios in cases and controls** |
|---|---|---|---|---|---|
| Control | 1.00 | 1.00 | 1.00 | | 1.00 |
| AML | 1.04 | 1.56 | 5.58 | 0.022 | 1.20 |
| | (0.61–1.75) | (0.74–3.29) | (1.40–22.3) | | (1.07–1.33) |
| ALL | 0.61 | 2.47 | 3.76 | 0.121 | 1.11 |
| | (0.23–1.61) | (0.90–6.82) | (0.41–34.9) | | (0.99–1.24) |
| RAEB[c] | 1.62 | 1.64 | – | – | 1.05 |
| | (0.77–3.43) | (0.52–5.21) | | | (0.94–1.18) |
| CML | 0.67 | 1.89 | 4.92 | 0.102 | 1.02 |
| | (0.32–1.38) | (0.82–4.35) | (0.85–28.5) | | (0.92–1.14) |

[a]Adjusted for sex, age (five groups), study centre and instruction. [b]Titres ≤ 40 have been used as reference group. [c]Four groups of age. Abbreviations as in Table 1.

compared with controls at higher serum dilutions (OR = 5.58, 95% CI 1.40–22.3) (Table 3); similar results were obtained when GMT ratios were analysed. In addition, the OR increased with increasing seroreactivity in the IFA assay (*P* for trend = 0.022).

### DISCUSSION

Our present study, the largest serological case-control investigation conducted on patients with haematologic malignancies who had not yet undergone immunosuppressive chemotherapy, identified a slight significant association between increased HHV-6 seropositivity and GMT ratio with AML (*P* for trend = 0.022, adjusted OR 1.20, 95% CI 1.07–1.33) respectively. The results presented here are in agreement with those obtained in a case-control study in which the prevalence of antibodies to HHV-6 in AML patients was higher than in the control group: GMT = 2.66, 95% CI 1.90–3.74 (Clark et al, 1990). Furthermore, two cases of monoblastic leukaemia were reported in which malignant cells contained HHV-6 DNA, antigens and immature viral particles (Krueger et al, 1994), whereas, in another study, HHV-6 DNA was undetectable by blot hybridization in ten AML patients (Josephs et al, 1988).

The negative results regarding an association between HHV-6 antibody titers and ALL, CML, or RAEB in the present study are also consistent with the literature. Ablashi et al (1988*b*) found higher levels of HHV-6 antibodies in a small group of children with ALL as compared with normal subjects. However, Levine et al (1992*a*) found no significant differences in antibody titers between 50 patients with ALL and 50 blood donors. This last finding suggests that the association previously reported for HHV-6 and ALL was a result of the study population age, rather than a true relationship.

Seroepidemiological evidence indicates that HHV-6 infection (IgG antibody titres of > 1:20 by IFA) is common and widespread, with a proportion of subjects ranging from 20% to 80% (Okuno et al, 1989; Levy et al, 1990; Yanagi et al, 1990). In this study, 18.2% of the control population and an average of 22.3% (range 15.1–25.9%) of the cases were seropositive at 1:80 serum dilution by IFA. This positivity rate is actually lower than those previously reported (Okuno et al, 1989; Levy et al, 1990; Yanagi et al, 1990; Torelli et al, 1991). Furthermore, in Italy, Torelli et al (1991) found antibodies against HHV-6 in 54% of 200 blood donors at a 1:40 serum dilution. The differences found in HHV-6 seroprevalence may result from the use of different serological assays and criteria of positivity. The majority of the epidemiological studies were performed with IFA, but the limiting titre for seropositivity varied. The limiting titre adopted in this study was 80, which includes 82% of the control population.

Other factors that may account for differences in HHV-6 seroprevalence include the definition of the control population: there is a possibility that HHV-6 is transmitted by the transfusion of blood or blood components (Wilborn et al, 1994). We excluded those patients who received blood product transfusions from both cases and controls. The possibility that differences in antibody titres could be detected using different isolates remains to be investigated. In a previous study on HHV-6 infection in Italy no variations in seroprevalence or in antibody GMT were observed assaying the sera against the strains G.S., U1102, or Z29 infected cells (Ragona et al, 1994).

Several studies conducted in healthy individuals showed that HHV-6 seroprevalence can be influenced by age, sex and geographic/ethnic origin, titres being higher in children than in adults, in females than in males and Ghanaians compared with Asians and White Americans (Levine et al, 1992*b*; Ragona et al, 1994; Braun et al, 1997). In our study no differences in seropositivity were observed on the basis of age or sex.

In cancer patients, therapy may play a role in antibody titres, and the high levels of HHV-6 antibody may be a result of therapy rather than relating to the neoplastic disease. The possibility of reactivation of HHV-6 infection by therapy-induced immunosuppression can be excluded because sera were drawn at the time of diagnosis, i.e. well before chemotherapy was administered. In addition, none of the cases studied here had received previous treatments for past malignancies. Furthermore, IFA reactivity was not elevated among cases, as might be expected if there was a general activation of HHV-6 infection. However, because the comparison was performed between values obtained at 1:80 and higher serum dilutions, we conclude that the titre of anti-HHV-6 antibodies and not the frequency of infection is significantly higher in AML individuals than in control subjects. This finding is not clear; however, serology data indicated that immune system of AML patients is activated against HHV-6 antigens. Furthermore, the serological assays currently used for identifying HHV-6 infection do not differentiate between persistent, latent or active

© 1999 Cancer Research Campaign infections. Detection of HHV-6 DNA in serum or plasma has been shown to be a marker of active infection (Secchiero et al, 1995), but does not clarify if HHV-6 has a possible casual role or viral reactivation is triggered by the illness itself. In conclusion, a slight significant association between HHV-6 antibody titres and AML patients not previously treated was observed, while no significant association was found between HHV-6 antibodies and ALL, RAEB, or CML. Further studies should be performed to further explore the relationship between HHV-6 infection and AML leukaemias.

### ACKNOWLEDGEMENTS

Preliminary data from this study were presented at the 87th Annual Meeting of American Cancer Association for Cancer Research, held in Washington, DC, in April 1996 (Abst. 1741).

This study was partially supported by Italian Ministry of Health, Grant 500.4/RSC/70.18/T/1886; by the 'Consiglio Nazionale delle Ricerche' Bilateral Project Italia–USA Grants 87.00186.04, 88.00600.04, and 89.02995.04; by 'Progetto Finalizzato' ACRO 'Consiglio Nazionale delle Ricerche', Grant 92.02219.39; and by AIRC ('Associazione Italiana Ricerca Cancro').

### REFERENCES

Ablashi DV, Lusso P, Hung C-L, Salahuddin SZ, Josephs SF, Llana T, Kramarsky B, Biberfeld P, Markham PD and Gallo RC (1988*a*) Utilization of human hematopoietic cell lines for the propagation and characterization of HBLV (Human Herpesvirus 6). *Int J Cancer* **42**: 787–791

Ablashi DV, Josephs SF, Buchbinder C, Hellman K, Nakamura S, Llana T, Lusso P, Kaplan M, Dahlberg J, Memon S, Iman F, Ablashi KL, Markham PD, Kramarsky B, Krueger GRF, Biberfeld P, Wong-Staal F, Salahuddin SZ and Gallo RC (1988*b*) Human B-lymphotropic virus (human herpesvirus 6). *J Virol Methods* **21**: 29–48

Braun DK, Pellett PE and Hanson CA (1995) Presence and expression of human herpesvirus 6 in peripheral blood mononuclear cells of S100-positive, T-cell chronic lymphoproliferative disease. *J Infect Dis* **171**: 1351–1355

Braun DK, Dominguez G and Pellet PE (1997) Human herpes virus 6. *Clin Microbiol Rev* **10**: 521–567

Clark DA, Alexander FE, McKinney PA, Roberts BE, O'Brien C, Jarrett RF, Cartwright RA and Onions DE (1990) The seroepidemiology of human herpesvirus-6 (HHV-6) from a case-control study of leukaemia and lymphoma. *Int J Cancer* **45**: 829–833

Di Luca D, Dolcetti R, Mirandola P, De Re V, Secchiero P, Carbone A, Boiocchi M and Cassai E (1994) Human herpesvirus 6: a survey of presence and variant distribution in normal peripheral lymphocytes and lymphoproliferative disorders. *J Infect Dis* **170**: 211–215

Josephs SF, Buchbinder A, Streicher HZ, Ablashi DV, Salahuddin SZ, Guo H-G, Wong-Staal F, Cossman J, Raffeld M, Sundeen J, Levine P, Biggar R, Krueger GRF, Fox RI and Gallo RC (1988) Detection of human B-lymphotropic virus (human herpesvirus 6) sequences in B cell lymphoma tissues of three patients. *Leukemia* **2**: 132–135

Krueger GRF, Klueppelberg U, Hoffmann A and Ablashi DV (1994) Clinical correlates of infection with human herpesvirus-6. *In Vivo* **8**: 457–486

Levine PH, Ablashi DV, Saxinger WC and Connelly RR (1992*a*) Antibodies to human herpes virus-6 in patients with acute lymphocytic leukemia. *Leukemia* **6**: 1229–1231

Levine PH, Neequaye J, Yadav M and Connelly R (1992*b*) Geographic/ethnic differences in human herpesvirus-6 antibody patterns. *Microbiol Immunol* **36**: 169–172

Levy JA, Ferro F, Greenspan D and Lennette ET (1990) Frequent isolation of HHV-6 from saliva and high seroprevalence of the virus in the population. *Lancet* **i**: 1047–1050

Luka J, Pirruccello SJ and Kersey JH (1991) HHV-6 genome in T-cell acute lymphoblastic leukemia. *Lancet* **338**: 1277–1278

Luppi M, Marasca R, Barozzi P, Ferrari S, Ceccherini-Nelli L, Batoni G, Merelli E and Torelli G (1993) Three cases of human herpesvirus-6 latent infection: integration of viral genome in peripheral blood mononuclear cell DNA. *J Med Virol* **40**: 44–52

Mele A, Szklo M, Visani G, Stazi MA, Castelli G, Pasquini P, Mandelli F and the Italian Leukemia Study Group (1994) Hair dye use and other risk factors for leukemia and pre-leukemia: a case-control study. *Am J Epidemiol* **139**: 609–619

Okuno T, Takahashi K, Balachandra K, Shiraki K, Yamanishi K, Takahashi M and Baba K (1989) Seroepidemiology of human herpesvirus 6 infection in normal children and adults. *J Clin Microbiol* **27**: 651–653

Ragona G, Calogero A, Cirone M, Cuomo L, Gonnella R, Zompetta C, Gentile G, Martino P, Menichella D, Frati L and Faggioni A (1994) HHV-6 infection in Italy: characterization of an endemic isolate and seroepidemiologic analysis. *Clin Diagn Virol* **1**: 261–270

Ragona G, Angeloni A, Farina A, Faggioni A, Frati L, Calogero A, Gentile G, Martino P, Arcese W and Mandelli F (1995) Subclinical infection of the respiratory tract of immunocompromised patients by human herpesvirus-6. *Blood* **85**: 295–296

Razzaque A (1990) Oncogenic potential of human herpesvirus-6 DNA. *Oncogene* **5**: 1365–1370

Razzaque A, Williams O, Wang T and Rhim JS (1993) Neoplastic transformation of immortalized human epidermal keratinocytes by two HHV-6 DNA clones. *Virology* **195**: 113–120

Salahuddin SZ, Ablashi DV, Markham PD, Josephs SF, Sterzenegger S, Kaplan M, Halligan G, Biberfeld P, Wong- Staal F, Kramarsky B and Gallo RC (1986) Isolation of a new virus, HBLV, in patients with lymphoproliferative disorders. *Science* **234**: 596–601

Secchiero P, Carrigan DR, Asano Y, Benedetti L, Crowley RW, Komaroff AL, Gallo RC and Lusso P (1995) Detection of human herpesvirus 6 in plasma of children with primary infection and immunosuppressed patients by polymerase chain reaction. *J Infect Dis* **171**: 273–280

Thompson J, Choudhury S, Kashanchi F, Doniger J, Berneman Z, Frenkel N and Rosenthal LJ (1994) A transforming fragment within the direct repeat region of human herpesvirus type 6 that transactivates HIV-1. *Oncogene* **9**: 1167–1175

Torelli G, Marasca R, Luppi M, Selleri L, Ferrari S, Narni F, Mariano MT, Federico M, Ceccherini-Nelli L, Bendinelli M, Montagnani G, Montorsi M and Artusi T (1991) Human herpesvirus-6 in human lymphomas: identification of specific sequences in Hodgkin's lymphomas by polymerase chain reaction. *Blood* **77**: 2251–2258

Wilborn F, Schmidt CA, Zimmermann R, Brinkmann V, Neipel F and Siegert W (1994) Detection of herpesvirus type 6 by polymerase chain reaction in blood donors: random tests and prospective longitudinal study. *Br J Haematol* **88**: 187–192

Yanagi K, Harada S, Ban F, Oya A, Okabe N and Tobinai K (1990) High prevalence of antibody to human herpesvirus-6 and decrease in titer with increase in age in Japan. *J Infect Dis* **161**: 153–154

*© 1999 Cancer Research Campaign*

